# Evaluation of the viability and functionality of human peripheral blood mononuclear cells cryopreserved up to 2 years in animal-protein-free freezing media compared to the FBS-supplemented reference medium

**DOI:** 10.3389/fimmu.2025.1627973

**Published:** 2025-08-01

**Authors:** Frédérique Jantet-Blaudez, Joseline Ruiz, Sylviane Gautheron, Anke Pagnon

**Affiliations:** ^1^ Translational Immunology, Vaccines R&D, Sanofi, Marcy l'Etoile, France; ^2^ Translational and Early Development Biostatistics, Vaccines R&D, Sanofi, Marcy l'Etoile, France

**Keywords:** PBMC cryopreservation, serum-free media, cell viability, animal-protein-free media, biobanking optimization

## Abstract

**Background:**

Cryopreservation of peripheral blood mononuclear cells (PBMCs) is crucial for consistent analysis in immunological studies and clinical trials. Traditional freezing media often contain fetal bovine serum (FBS), which raises ethical concerns and the risk of pathogen transmission, and dimethyl sulfoxide (DMSO), known for its cytotoxic effects. This study evaluates the viability, yield, phenotype, and functionality of PBMCs cryopreserved in several commercially available animal-protein-free media, some with reduced or no DMSO, compared with a reference medium over 2-years.

**Methods:**

PBMCs from 11 healthy volunteers were cryopreserved in a reference medium (FBS + 10% DMSO) and nine alternative serum-free media with varying DMSO concentrations. Cell viability and functionality were assessed at 3 weeks (M0), 3 months (M3), 6 months (M6), 1 year (M12), and 2 years (M24) post-freezing. Media with DMSO concentrations below 7.5% were excluded after M0 due to lower viability. Cell functionality was assessed using various assays including cytokine secretion profiles, T and B FluoroSpot, and intracellular cytokine staining.

**Results:**

PBMCs cryopreserved in CryoStor CS10 and NutriFreez D10 maintained high viability and functionality, comparable to the FBS10 reference medium, across all time points. Bambanker D10 displayed a comparable viability but tended to diverge from the FBS10 reference medium in terms of T cell functionality. CryoStor CS7.5, while showing promising results, was eliminated due to being a mixture of CS10 and CS5, which could potentially introduce errors in preparation. Media with < 7.5% DMSO showed significant viability loss and were eliminated after the initial assessments. Serum-free media with 10% DMSO, comparable to FBS-based media, effectively preserved PBMC immune response.

**Conclusions:**

CS10 and NutriFreez D10, both serum-free media containing 10% DMSO, are viable alternatives to FBS-based media for the long-term cryopreservation of PBMCs. These media ensure comparable cell viability and functionality, supporting their application in clinical and research settings to address the important drawbacks associated with FBS use.

## 1 Introduction

Peripheral blood mononuclear cells (PBMCs) play a crucial role in clinical studies on vaccines and immune-based therapies (1). Isolated from blood samples of study participants, PBMCs are essential for examining the immune response to interventions by assessing changes in antigen-specific T cell or B cell responses. However, once blood samples are collected, PBMCs must be isolated and promptly analyzed, as their viability and functionality decline quickly (2). This implies that, to ensure optimal analysis of functionality, PBMC samples must be analyzed both close to the place where blood was collected and soon after collection.

Cryopreserving samples allows circumventing this limitation. It allows the storage of test samples for later use, thus helping to avoid logistical challenges associated with testing of fresh samples. The possibility to freeze PBMC samples enables the inclusion of sites in a clinical trial that would otherwise be off-limits due to the lack of adequate testing facilities. Frozen samples can be shipped and stored at a central laboratory for subsequent testing, helping to decrease variability that would ensue from analyses being performed in different facilities. Regardless of a study’s enrollment pace, the number of participants, the number of clinical sites involved, or the interval between time points, cryopreserved samples can be tested according to a set experimental plan. Importantly, samples collected from a study participant at different time points can be analyzed simultaneously, further reducing potential variability in the results.

Nowadays, following developments in the cryopreservation of other cell types over decades, the viability and functionality of PBMCs can be extended through freezing, making it possible to build repositories of a large number of samples (3, 4). Freezing of PBMCs is especially important for Phase I and II vaccine studies, in which the cellular immune response of participants is investigated over different time points (1). Although cryopreservation of cells is crucial across a wide range of research areas, it exposes frozen cells to extreme conditions that may alter their functional and phenotypic characteristics. To limit the impact of freezing on these characteristics, cells are frozen in a specific medium. Optimal cryopreservation should allow maintaining the quantity, quality, phenotype, and functionality of the cells (1, 2, 5).

Currently, the most commonly used freezing media for PBMC cryopreservation consist of fetal bovine serum (FBS) supplemented with 10% dimethyl sulfoxide (DMSO) as the cryoprotectant. DMSO strong membrane permeability (6) stabilizes the cell membrane, thus preventing a strong osmotic shock (4). This combination is known for efficiently protecting cells, but there are several challenges associated with these components. Firstly, while DMSO is effective in preventing ice crystal formation during freezing, it exhibits cytotoxicity at room temperature. Minimizing cell exposure to it during the freezing and thawing process can improve cell viability.

Secondly, FBS in cell culture or freezing media can potentially transmit infectious agents to the cells, and specifically for PBMCs, can induce unwanted immunological responses during cell culture, which may affect experimental outcomes. Each FBS batch is unique and requires qualification before being used to assess the presence of undesirable components, such as hormones, growth factors, endotoxins, or microorganisms, such as viruses or mycoplasma, that could interfere with the measurement of immunological responses (7, 8).

Additionally, much like the use of animals in non-clinical safety and toxicology studies, supplementing cell culture and freezing media with FBS raises ethical questions, providing a strong rationale for seeking replacement solutions. These ethical concerns have prompted many academic and pharmaceutical groups to seek animal-protein-free alternatives (9, 10). Finally, from a logistic perspective, import restrictions in certain countries can make FBS acquisition problematic, if not entirely unfeasible (8). These multifaceted challenges underscore the compelling reasons to transition away from FBS-based freezing media in PBMC cryopreservation.

In light of these challenges, this study was designed to systematically evaluate alternative cryopreservation media for PBMCs. We conducted a comprehensive comparison between several commercially available serum-free freezing media and the traditional FBS+10% DMSO reference medium based on several biological criteria, including viability, cell yield, and cell phenotypes, as well as the functionality of T and B cells. Furthermore, we sought to address the cytotoxicity concerns associated with DMSO by investigating the feasibility of reducing its concentration in the freezing medium without compromising the stability of PBMCs over a long cryopreservation period. While previous studies have examined the effect of cryopreservation media on the viability of PBMCs and their T cell functionality, comparing xeno-free media and/or DMSO-free media with FBS+10% DMSO (1, 11–13), these comparisons were typically made over shorter periods or did not involve multiple time points. To the best of our knowledge, no studies have specifically investigated the viability and functionality of PBMCs following cryostorage for a period as long as 2 years.

## 2 Methods

### 2.1 Sample collection, processing and cryopreservation

The freezing media for this study were selected based on several practical criteria essential for clinical trial implementation: 1) commercial availability and ease of procurement, 2) manufacturer’s stability claims, 3) ease of use in clinical settings, 4) ready-to-use formulations, and 5) storage requirements compatible with clinical site capabilities. These criteria ensure that the selected media could be readily adopted in real-world clinical trial scenarios.

**Figure 1:**
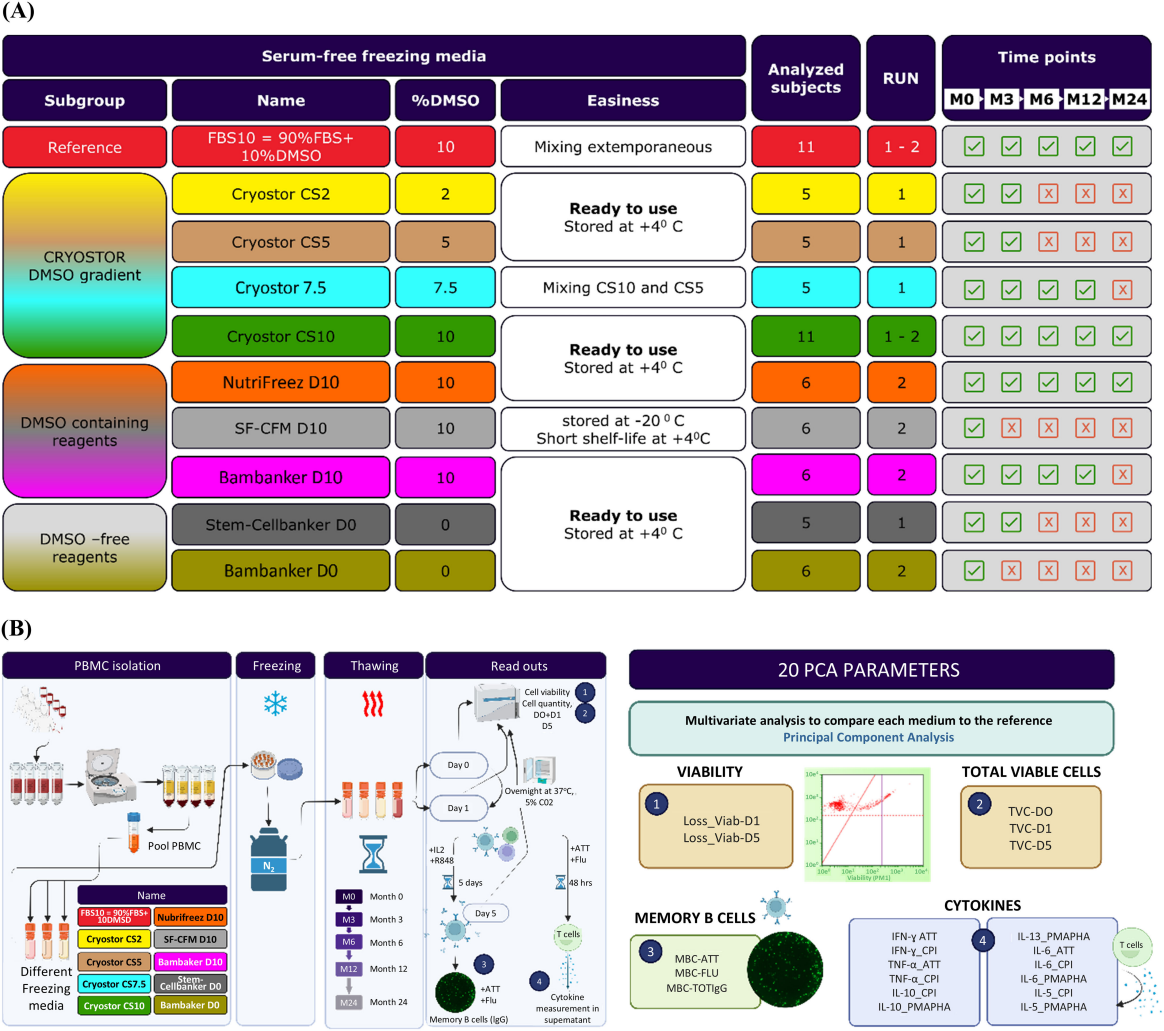
Study design and experimental workflow. **(A)** All the nine commercial freezing media were without FBS, except the reference FBS10. They were classified into three subgroups: the CryoStor family of media with a DMSO gradient from 10% to 0%, media containing 10% DMSO, and DMSO-free media. The first run included samples from five subjects frozen in reference FBS10, CryoStor media, and Stem-Cellbanker D0. The second run included samples from six other subjects frozen in reference FBS10, CryoStor CS10 (best performing medium of the first run), three other media containing 10% DMSO, and the Bambanker D0. As illustrated in the Time points column, evaluation of some media was discontinued after the first, second and fourth time points due to significant viability loss (Bambanker D0, Stem-Cellbanker D0, Cryostor CS2, Cryostor CS5), practical limitations (SF-CFM D10, CS7.5), and difference with the reference medium in terms of T cell functionality (Bambanker D10). **(B)** The nine animal- protein-free freezing media were compared to the FBS+10% DMSO through biological criteria like viability, cell recovery rate, and T and B cell functionality. Viability and cell recovery rate were evaluated at D0 after thawing, at D1 after an overnight resting and at D5 after a polyclonal B cell stimulation. Functional assays were performed only at D1 after an overnight resting. For this purpose, PBMC from eleven volunteers were purified and frozen in the reference media and the 9 other media and the biological parameters described above were assessed at regular intervals up to 2 years of freezing. A multivariate statistical analysis called PCA was performed at each time point and between timepoints to compare the reference medium to the freezing media under evaluation. This figure was generated using BioRender software.

To evaluate various cryopreservation media (**Figure 1A**), whole blood (450–480 mL) from 11 healthy volunteers without known antigenicity was collected into standard blood bags by the French blood bank, Etablissement Français du Sang (EFS). PBMCs were isolated using a lymphocyte density gradient medium (Lymphoprep™, STEMCELL Technologies, Vancouver, Canada) and subsequently washed in Hanks’ Balanced Salt Solution buffer. Prior to the final centrifugation, the cell solution was divided into different tubes to separate and resuspend cell pellets in each cryopreservation medium being tested (**Figure 1B**). These included the reference freezing medium (FBS10, composed of 90% FBS (Hyclone, #SH30084, Cytiva, Marlborough, MA, USA) and 10% DMSO (Sigma-Aldrich, #D2650, St. Louis, MO, USA) and nine alternative media formulations: CryoStor CS2 (#07932), CS5 (#07933), CS7.5 (mix of CS10 and CS5), and CS10 (#100-1061), all from STEMCELL Technologies (Vancouver, Canada), with DMSO concentrations of 2%, 5%, 7.5%, and 10%, respectively; NutriFreez^®^ D10 (#01-0031-100, Tebu Bio, Le Perray-en-Yvelines, France), SF-CFM D10 (#0143, ScienCell Research Laboratories, Carlsbad, CA, USA), Bambanker™ D10 (#BB02, GC Lymphotec, Tokyo, Japan), all with 10% DMSO; and DMSO-free options such as Stem-Cellbanker^®^ D0 (#11924, AMSBIO, Abingdon, UK) and Bambanker™ D0 (#BBF01, GC Lymphotec, Tokyo, Japan). For each medium, seven aliquots of 1 mL cell suspension, at a concentration of 12 × 10^6^ cells/mL, were dispensed into pre-cooled cryovials, transferred to CoolCell^®^ (BioCision, San Rafael, CA, USA) containers, and placed into a −80°C freezer for 1–7 days before transfer to vapor-phase liquid nitrogen storage. PBMCs from each donor were evaluated at five time points: 3 weeks (M0), 3 months (M3), 6 months (M6), 1 year (M12), and 2 years (M24) post-freezing. The samples were processed in two separate runs, with the second run beginning 3 months after the first to incorporate the best-performing medium from the initial run based on M0 results.

### 2.2 Thawing PBMCs

To assess the impact of cryopreservation, we used a standardized thawing protocol for all samples. At each time point, one cryovial containing 1 mL of cell suspension from each donor was thawed by gently agitating the vial in a +37°C water bath until the cell suspension completely melted. Following thawing, a mixture of FBS and deoxyribonuclease I (DNase, #11284932001, Roche Diagnostics, Mannheim, Germany) at a concentration of 10 µg/mL was added to the vial. The entire cell suspension was then transferred into 10 mL of prewarmed (+37°C) RPMI 1640 (#31870074, Gibco, Thermo Fisher Scientific, Waltham, MA, USA) medium. This medium was supplemented with 10% FBS, 200 mM L-glutamine (#25030081, Gibco, Thermo Fisher Scientific, Waltham, MA, USA), and penicillin-streptomycin (10,000 U/mL penicillin, 10,000 µg/mL streptomycin, #15240-096, Gibco, Thermo Fisher Scientific, Waltham, MA, USA). The PBMCs were washed twice by centrifugation before proceeding with cell counting.

### 2.3 Determination of PBMC viability and recovery

**Figure 2:**
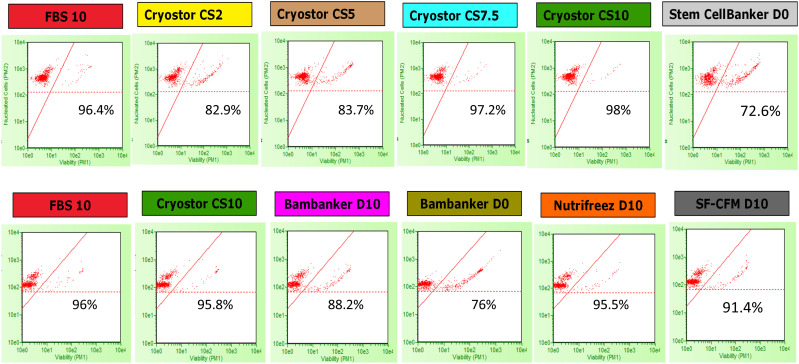
Examples of cell count on the Guava^®^ Cytometer at M0 (D1). Viability and total nucleated cell count analysis of cells stained with ViaCount. Cells were cryopreserved in each of the 10 cryopreservation media. Viability percentages for each condition are indicated within each respective plot. The *y*-axis represents the nucleated cell count (in arbitrary units), and the *x*-axis indicates cell viability (measured by propidium iodide [PI] and viability dye exclusion). Red dots represent individual cells, the red diagonal line shows the viability threshold, and the horizontal dotted red line depicts the cut-off for nucleated cells. The first and second rows present cell count from a single donor in Run 1 and a different donor in Run 2, respectively, at the M0 time point, after thawing and following an overnight resting period (D1).

We evaluated PBMC viability and recovery rates to determine the immediate effects of different cryopreservation media. PBMC viability and recovery were assessed using the Guava^®^ ViaCount™ reagent (SKU4000-0040, Cytek Biosciences, Fremont, CA, USA), which distinguishes viable, apoptotic, and dead cells based on membrane integrity using two DNA-binding dyes: a nuclear dye for nucleated cells and a viability dye for dying cells. Debris was excluded due to their lack of staining. Stained PBMCs were analyzed on a Guava^®^ Cytometer 5HT_L (Cytek). The dot plot displayed nucleated cell counts (*y*-axis) and cell viability (*x*-axis), with red dots representing individual cells, and thresholds distinguishing viable from non-viable cells and background noise (**Figure 2**). Viability and cell numbers were measured at time points M0, M3, M6, M12, and M24 immediately after thawing (D0), after overnight resting at +37°C (D1), and after 5 days of polyclonal stimulation (D5) (**Figure 1B**). The loss of viability was calculated as the difference between viability percentages at D1 and D0 (Loss_Viab_D1) and between D5 and D1 (Loss_Viab_D5). Viable cell recovery was determined by calculating the percentage of cells recovered at each stage relative to the previous time point: D0 relative to D-1 (i.e., before freezing), D1 relative to D0, and D5 relative to the number of cells put into culture at D1.

### 2.4 PBMC phenotyping

Two years post-freezing, cell phenotyping was performed to evaluate the potential impact on the different cell populations involved in the immunological response. A 14-color phenotyping kit (#R7-40000, Cytek Biosciences, Fremont, CA, USA) was used to label the following cell surface markers: CD3, CD4, CD8, CD14, CD16, CD19, CD25, CD27, CCR7, IgD, CD45, CD45RA, CD56, and CD127. A viability marker (ViaDye Red, #R7-60008, Cytek Biosciences, Fremont, CA, USA) was included to assess cell viability.

**Figure 3:**
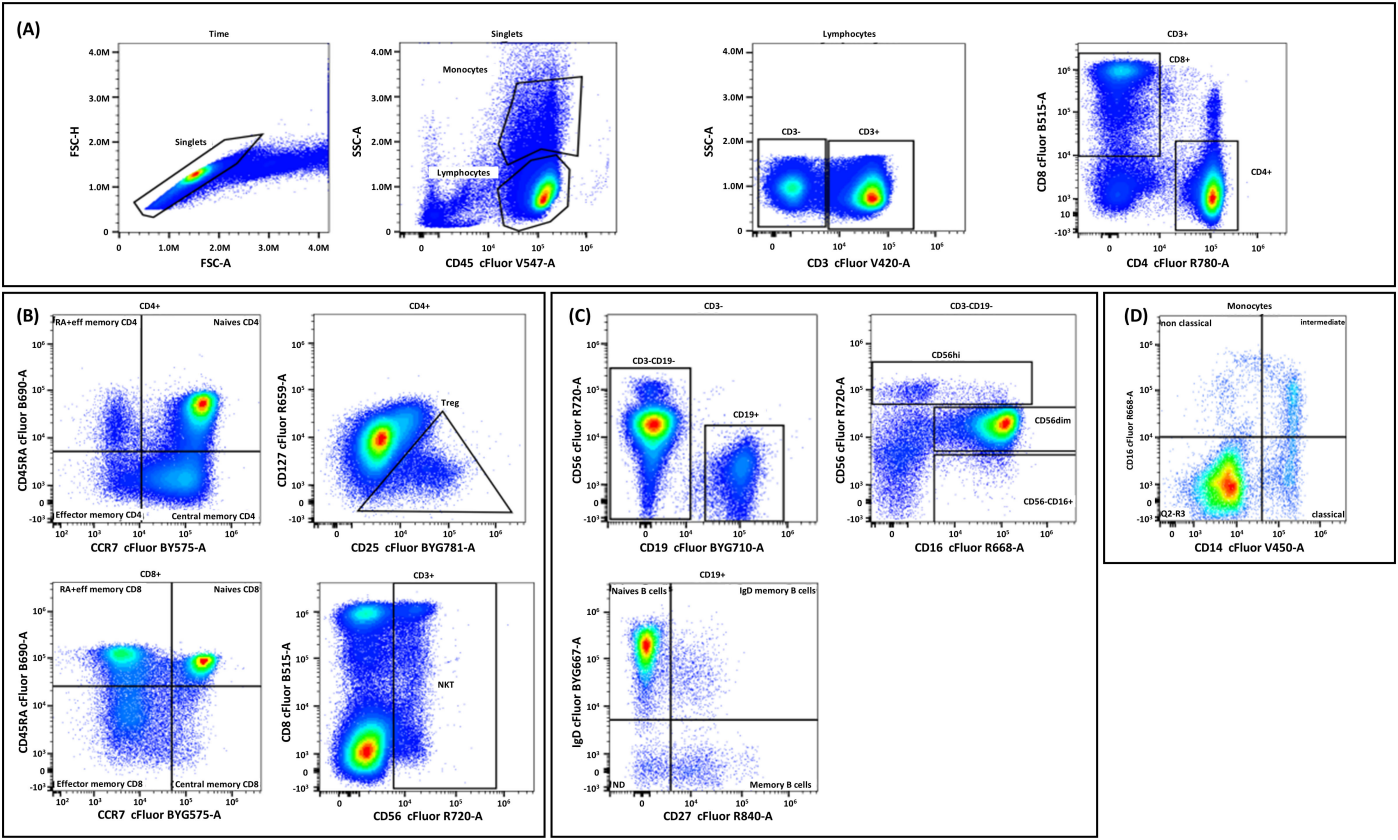
Flow cytometry gating strategy and analysis of different cell populations. Initially, time versus Forward Scatter Area (FSC-A) was plotted to exclude debris, followed by selecting singlet cells on FSC-A versus Side Scatter Area (SSC-A) to eliminate doublets. **(A)** Lymphocytes and monocytes were identified on a CD45 versus SSC-A plot. **(B)** For T cell analysis, CD3+ cells were first identified and divided into CD4+ and CD8+ subsets. CD4+ T cells were further classified into naïve, central memory, effector memory, and RA+ effector memory subsets, with regulatory T cells identified by CD25 and CD127 expression. Similarly, CD8+ T cells were subdivided into naïve and memory subsets. Natural killer (NK) T cells were identified within the CD3+ population. **(C)** B cells (CD19+) were divided into naïve, memory, and IgD memory subsets based on IgD and CD27 expression. NK T cells were identified within the CD3-CD19- population and further classified into CD56high, CD56dim, and CD56−CD16+ subsets based on CD56 and CD16 expression. **(D)** Monocytes were further categorized into nonclassical, intermediate, and classical subsets based on CD16 and CD14 expression.

PBMCs were distributed in a 96-well plate at 10^6^ cells/well in PBS. Cells were incubated with ViaDye Red (1/10,000 in PBS) for 20 min at room temperature, washed twice with Dulbecco’s PBS (DPBS) supplemented with 0.5% BSA, and then incubated with 5 µL of Fc receptor blocking solution (Human TruStain FcX™ block, #422302, BioLegend, San Diego, CA, USA) for 15 min. The 14 Cytek markers were added sequentially: 5 µL of IgD for 10 min, 5 µL of CCR7 for 10 min, and the remaining markers for 30 min at room temperature. After washing twice with DPBS + 0.5% BSA, cells were distributed at 200 µL/well for spectral cytometry on an Aurora platform (Cytek). Flow cytometry data analysis was performed using a hierarchical gating strategy to identify major cell populations and their subsets. The detailed gating strategy is illustrated in **Figure 3**.

### 2.5 Memory B cell fluorospot assay

At D1, PBMCs were cultured for 5 days at +37°C with 5% CO_2_ at a concentration of 10^6^ cells/mL in complete RPMI 1640 medium, supplemented with recombinant human IL-2 (10 ng/mL) and toll-like receptor 7 agonist R-848 (1 µg/mL) from Mabtech (StimPack, #3652-1, Mabtech, Stockholm, Sweden) to induce the terminal differentiation of memory B cells into antibody-secreting cells.

FluoroSpot PVDF plates (Mabtech) were pre-wetted with 35% ethanol (25 µL/well), washed with phosphate‐buffered saline (PBS), and coated overnight with Tetanus toxoid (TT, produced in-house) and a mixture of three influenza vaccine strains (TIV; produced in-house, 5 µg/mL each) to assess specific immunoglobulin G (IgG) responses. Separate wells were coated with an anti-IgG capture monoclonal antibody (15 µg/mL; anti-human IgG Mab MT145 unconjugated, #3850-1-250, Mabtech, Stockholm, Sweden) to assess total IgG-secreting memory B cells. The plates were then washed with PBS and blocked with complete RPMI for 2.5 hours at +37°C.

Stimulated PBMCs were washed twice by centrifugation, counted on the Guava^®^ counter and resuspended in complete RPMI at 4 × 10^6^ cells/mL. Cells were added in duplicate to coated plates (100 µL/well) at serial quantities: 400,000, 200,000, and 100,000 cells/well for specific responses, and 10,000, 5,000, and 2,500 cells/well for total IgG response. The plates were incubated for 5 h at +37°C with 5% CO_2_, washed with PBS, and exposed to the anti-IgG-550 (Cy3 fluorochrome filter) fluorescent secondary antibody (Detection Mab anti-human IgG-550 MT78/145, Mabtech, Stockholm, Sweden) for 2 hours at +20°C. After washing and drying, spots were counted with a FluoroSpot reader (IRIS, Mabtech, Stockholm, Sweden).

### 2.6 PBMC stimulation for cytokine measurement

At D1, PBMCs from each donor were cultured in 96-well plates at +37°C with 5% CO_2_ at a concentration of 2 × 10^6^ viable cells/mL. The cells were either left unstimulated or subjected to various stimulation conditions for 48h. For polyclonal T cell activation, a mixture of 10 ng/mL Phorbol 12-Myristate 13-Acetate (PMA; #P8139, Sigma-Aldrich, St. Louis, MO, USA) and 1 µg/mL Phytohaemagglutinin (PHA; #R30852801, Remel Oxoid, Thermo Fisher Scientific, Waltham, MA, USA), was used. For specific T cell stimulation, TT antigen was used at the concentration of 5 µg/mL and a CPI solution (#CTL-CPI-001, Cellular Technology Limited, Cleveland, OH, USA) containing inactivated cytomegalovirus (CMV), parainfluenza, and influenza virions was used at 10 µg/mL. Cytokine levels (interferon (IFN)-γ, interleukin (IL)-5, IL-6, IL-10, IL-13, and tumor necrosis factor (TNF)-α) were measured using the Bio-Plex Pro Human Cytokine 17-Plex Panel (#M5000031YV, Bio-Rad Laboratories, Hercules, CA, USA), according to the manufacturer’s instructions, and expressed in picograms per milliliter (pg/mL).

### 2.7 IFN-γ/IL-4 FluoroSpot assay

The PBMCs were assayed for IFN-γ and IL-4 production in the presence of cytomegalovirus glycoprotein gB (CMV gB), trivalent influenza vaccine (TIV), PHA, or CPI. Pre-coated 96-well plates with anti-IFN-γ monoclonal antibody (mAb) (clone 1-D1K) and anti-IL-4 mAb (clone IL-4-I) (Fluorospot plus human IFN-γ/IL-4 kit, FSP-0116-2, Mabtech Stockholm, Sweden) were washed with PBS and blocked with complete RPMI for 2.5 hours at +37°C. After thawing, PBMCs were resuspended in the AIM V™ medium (Gibco 12055091 Thermo Fisher Scientific, Waltham, MA, USA) at 4 × 10^6^ cells/mL and added to wells (400,000 cells/well for the specific responses, 50,000 cells/well for the PHA response). Antigens were diluted in AIM V™ medium with human anti-CD28 and added at 1 µg/mL. Plates were incubated for 48 hours at +37°C with 5% CO_2_ and then washed with PBS.

Detection antibodies (anti-IFN-γ mAb 7-B6-1-BAM and biotinylated anti-IL-4 mAb) were added in PBS + 0.1% BSA and incubated for 2 hours at +20°C. After washing, FluoroSpot conjugates (anti-BAM-490 and SA-550, Mabtech) were added for 1 hour at +20°C. A fluorescence enhancer was added for 15 minutes. Plates were dried before counting spots with a FluoroSpot reader (IRIS, Mabtech).

### 2.8 Intracellular cytokine staining

A 25-color spectral flow cytometry panel was used to evaluate the impact of the freezing medium on rare antigen-specific T cells. After thawing, PBMCs were stimulated with peptides (CEFT MHC-II pool, #PM-CEFT-MHC-II-1 and CEFX MHC-II subset, #PM-CEFX-3, JPT Peptide Technologies, Berlin, Germany) or proteins (CPI, #CTL-CPI-001, Cellular Technology Limited, Cleveland, OH, USA or Pokeweed [Phytolacca americana] lectin, #L8777, Sigma-Aldrich, St. Louis, MO, USA) for 6 or 9 hours. Brefeldin A (#B7651, Sigma-Aldrich, St. Louis, MO, USA), Monensin (Golgi Stop containing monensin, #554724, BD Biosciences, San Jose, CA, USA), and PE-Cy5 mouse anti-human CD107a (clone H4A3, #555802, BD Biosciences, San Jose, CA, USA) were added at the time of stimulation. The stimulation was performed in the AIM V™ medium (Gibco), in 96-well plates incubated at +37°C, 5% CO_2_.

PBMCs were stained with live/dead blue fixable viability stain (#L34962, Molecular Probes, Eugene, OR, USA) in PBS, washed, and blocked with TruStain FcX block (#422302) and TruStain Monocyte Blocker (#426102); both from BioLegend (San Diego, CA, USA). Surface markers included antibodies from BioLegend (San Diego, CA, USA): Spark Blue 550 mouse anti-human CD3 (clone SK7, #344852) and PerCP mouse anti-human CD45 (clone 2D1, #368506); and from BD Biosciences (San Jose, CA, USA): BUV805 mouse anti-human CD4 (clone SK3, #612900), BUV395 mouse anti-human CD8 (clone RPA-T8, #563795), BV480™ mouse anti-human CD56 (clone NCAM16.2, #566124), BV480™ mouse anti-human CD14 (clone MϕP9, #566141), and BV480™ mouse anti-human CD19 (clone SJ25C1, #566103). These antibodies were mixed in brilliant stain buffer (#566385, BD Biosciences, San Jose, CA, USA) and incubated for 30 min.

After staining, cells were fixed and permeabilized with BD CytoFix/CytoPerm (#554722, BD Biosciences, San Jose, CA, USA), followed by intracellular staining with the following antibodies from BD Biosciences (San Jose, CA, USA) included: FITC mouse anti-human IFN-γ (clone B27, #554700), PE mouse anti-human MIP1β (clone D21-1351, #550078), R718 mouse anti-human IL-17A (clone N49-653, #566938), and BV510 mouse anti-human granzyme B (clone GB11, #563388). Antibodies from BioLegend (San Diego, CA, USA) included: BV605 mouse anti-human TNF-α (clone MAb11, #502936), BV786 mouse anti-human IL-2 (clone MQ1-17H12, #500348), APC mouse anti-human IL-4 (clone MP4-25D2, #500812), and APC-Fire 750 mouse anti-human Perforin (clone B-D48, #353318). These antibodies were incubated with the cells for 30 min in the dark.

**Figure 4:**
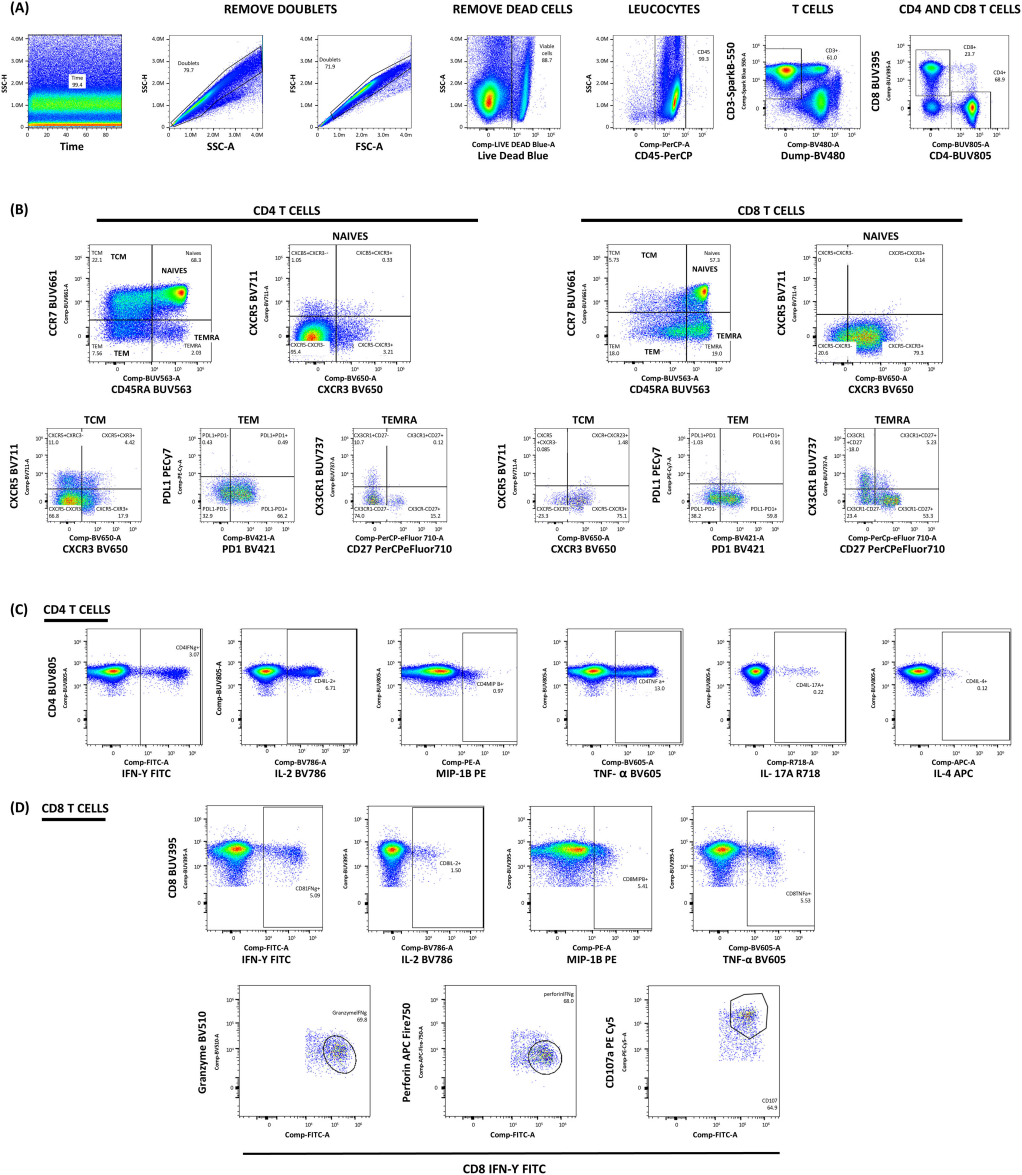
Gating strategy for the intracellular cytokine staining. **(A)** Initial gating strategy to identify viable T cell populations. Lymphocytes were identified based on Forward Scatter Area (FSC) and Side Scatter Area (SSC) properties, followed by doublet exclusion. Live CD45+ cells were selected after excluding dead cells using Live/Dead Blue. Within this population, T cells were identified as CD3+ cells, and a dump channel excluded CD14+CD19+CD56+ cells. **(B)** Characterization of T cell differentiation subsets. CD4+ and CD8+ T cells were further classified based on CCR7 and CD45RA expression into naïve T cells (CCR7+CD45RA+), central memory T cells (TCM, CCR7+CD45RA-), effector memory T cells (TEM, CCR7-CD45RA-), and terminally differentiated effector memory cells (TEMRA, CCR7-CD45RA+). Additional characterization included chemokine receptors (CXCR3, CCR5, CCR6, CXCR4, CXCR6), differentiation marker CD27, tissue-homing receptor CX3CR1, and immune checkpoint molecules CD279 (PD-1) and CD274 (PD-L1) **(C, D)** Analysis of antigen-specific T cell responses in CD4+ **(C)** and CD8+ **(D)** T cells. Within these defined T cell populations, functional responses were assessed by analyzing the expression of IFN-γ, CD107a, granzyme B, perforin, IL-2, IL-4, IL-17A, MIP-1β, and TNF-α following antigen stimulation.

Finally, the cell suspensions were washed twice and resuspended in wash buffer. Samples were analyzed on the Aurora Platform (five lasers) (#YAUV-50-B5-R5-V5-Y5, Cytek Biosciences, Fremont, CA, USA) with blue (488 nm), red (640 nm), violet (405 nm), yellow/green (561 nm), and UV (355 nm) lasers. Following the gating strategy, detailed in **Figures 4A–D**, analysis was performed using FlowJo 10.6.1 (Tree Star, Ashland, OR, USA).

### 2.9 One-sided Fisher’s exact test on ICS data

To compare the effects of different freezing media on T cell responses, intracellular cytokine staining (ICS) was used to detect rare subpopulations (0.01–0.1%) measuring antigen-specific cytokine production by CD4 and/or CD8 cells in response to *in vitro* stimulation of frozen human PBMCs in different media. Responders and non-responders were classified using a two-by-two contingency table comparing the number of positive and negative events in a certain gated population for both stimulated and unstimulated control samples.

A one-sided Fisher’s exact test was applied to each contingency table to test if the proportion of cytokine-producing cells in stimulated samples was equal to that in unstimulated controls. To account for multiple tests, a false discovery rate (FDR) adjustment was applied. Samples were considered responders if the adjusted *p*-value was inferior or equal to the significance level (*α*=0.001). The sum of the responders and non-responders to the *in vitro* stimulation was calculated for each medium (reference and new media) and is summarized in **Table 1**. For each medium comparison against the reference, four categories were observed: NR/NR (both non-responders), R/R (both responders), R/NR (reference responder, new medium non-responder), and NR/R (reference non-responder, new medium responder).

**Table 1:** Agreement between positive and negative responses between the different freezing media.

Media	NR/NR	NR/R	R/NR	R/R	Total
FBS10/CS10	137 (33)	9 (2)	8 (2)	262 (63)	416
FBS10/NF10	49 (31)	6 (4)	3 (2)	98 (63)	156
FBS10/StemCB	71 (34)	2 (1)	16 (8)	119 (57)	208

NR, non-responder; R, responder.

Data are presented as n (%).

### 2.10 Principal component analysis methodology

To compare different freezing media and identify a suitable substitute for the reference medium, principal component analysis (PCA) was used. PCA provided a summary and visual representation of the data through simple plots. Variables expressed in different units were centered at a mean of 0 and scaled to the standard deviation to ensure comparability.

For each experimental run and time point, PCA assessed the variance in cellular integrity and functionality data, including memory B and T cells quantification. This analysis covered 20 readouts for B, T, cell yield, and viability as well as 23 phenotyping readouts (**Table 2**). Assessments were conducted across 10 to 36 samples or conditions, representing combinations of PBMCs from six donors cryopreserved in three to six freezing media.

**Table 2:** List of readouts for each PCA.

Readout number	PCA phenotyping	PCA (cellular integrity and T and B cell functionality)
1	CD3pos	% cell recovery_D0
2	CD8pos	% cell recovery_D1
3	RApos_eff_memory_CD8	% cell recovery_D5
4	Naives_CD8	Loss_Viab_D1
5	Effector_memory_CD8	Loss_Viab_D5
6	memory_CD8	MBC_ATT (memory B cells)
7	CD4pos	MBC_VGT (memory B cells)
8	RApos_eff_memory_CD4	MBC_IgGTOT (memory B cells)
9	Naives_CD4	IFNg_ATT (T cells)
10	Effector_memory_CD4	IFNg_CPI (T cells)
11	Central_memory_CD4	TNFa_ATT (T cells)
12	NKT	TNFa_CPI (T cells)
13	Treg	IL13_PMAPHA (T cells)
14	CD3neg	IL5_CPI (T cells)
15	CD3negCD19neg	IL5_PMAPHA (T cells)
16	CD56hi	IL6_ATT (T cells)
17	CD56dim	IL6_CPI (T cells)
18	CD56negCD16pos	IL6_PMAPHA (T cells)
19	CD19pos	IL10_CPI (T cells)
20	Monocytes	IL10_PMAPHA (T cells)
21	M_non_classical	–
22	M_intermediate	–
23	M_classical	–

### 2.11 PARAFAC analysis

The PARAFAC (PARAllel FACtor) analysis extended the classical PCA for three-way data and the following three dimensions were included: donors, biological readouts, and time. The PARAFAC components summarized the interactions between these three modes.

## 3 Results

Our comprehensive investigation into alternative cryopreservation media followed a systematic approach, beginning with fundamental assessments of cell viability and recovery, progressing to detailed immunophenotyping, and culminating in functional analyses of specific immune cell populations.

### 3.1 PBMC recovery and viability

**Figure 5:**
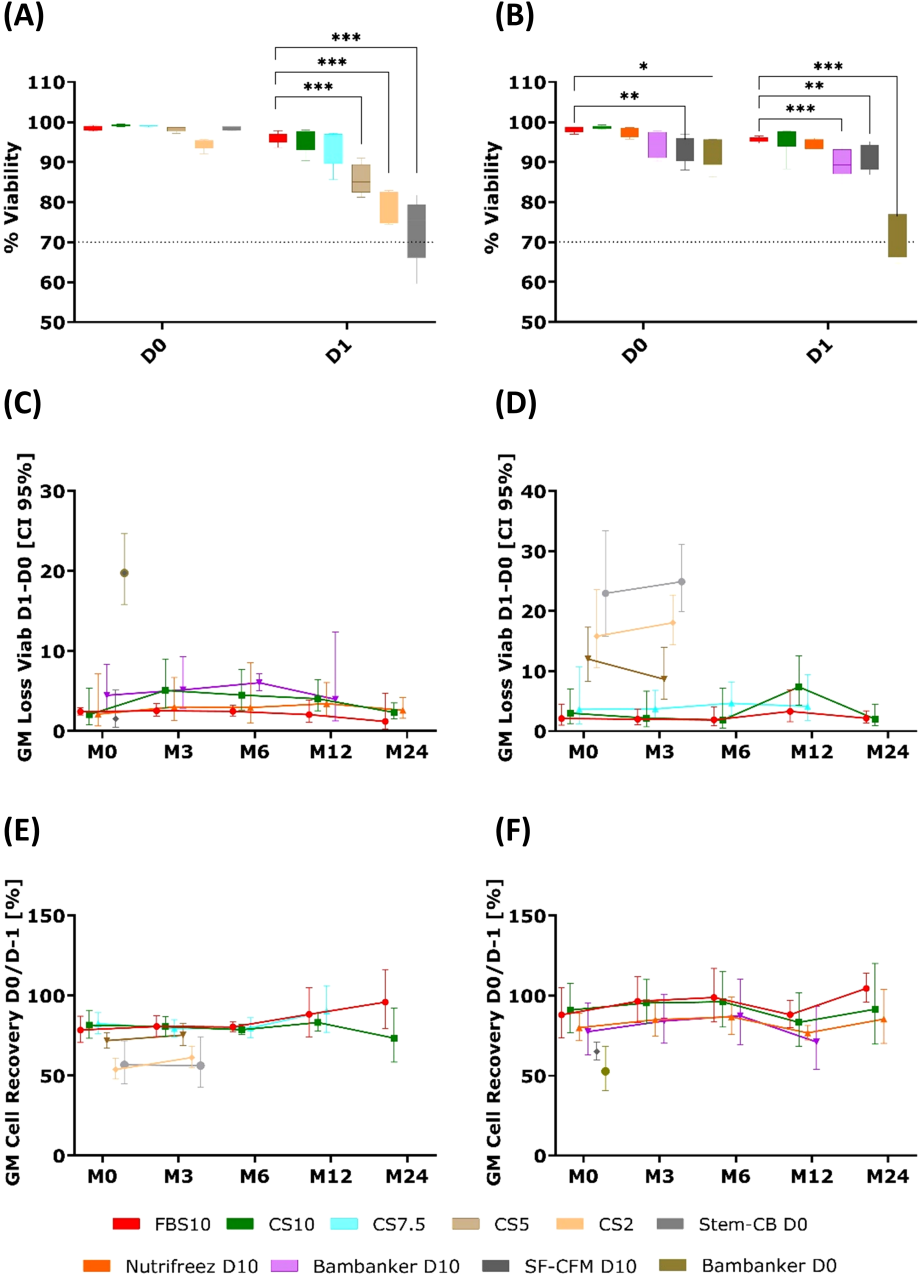
Comparable cell viability and recovery in PBMCs cryopreserved with FBS10, CS10, and NutriFreez D10, with reduced performance in low-DMSO and DMSO-free media. Comparison of PBMC viability and recovery across different cryopreservation media over 24 months. **(A, B)** Whisker plots showing viability percentages immediately after thawing (D0) and after overnight resting (D1) at M0 (3 weeks post-freezing) for Run 1 **(A)** and Run 2 **(B)**. The box extends from 25th to 75th percentiles with median line; whiskers show min to max values. Statistical comparison to FBS10 used a mixed model with medium and time as fixed factors, paired on donor, with Dunnett’s adjustment (*p < 0.05, **p < 0.01, ***p < 0.001). **(C, D)** Longitudinal analysis of geometric mean (GM) viability loss between D0 and D1 across time points (M0, M3, M6, M12, M24) for Run 1 **(C)** and Run 2 **(D)**. **(E, F)** Longitudinal analysis of GM cell recovery (percentage of cells recovered at D0 relative to pre-freezing count) across time points (M0, M3, M6, M12, M24) for Run 1 **(E)** and Run 2 **(F)**. Data from Run 1 (left panels, GM of five donors) and Run 2 (right panels, GM of six donors) are presented. Error bars represent 95% confidence intervals. It should be noted that evaluation of some media was discontinued after M0, M3, or M12.

The results presented in **Figure 5** show the impact of different cryopreservation solutions on PBMC viability loss and cell recovery over a 24-month period, across two independent experimental runs (Run 1 and Run 2). At the M0 time point (3 weeks post-freezing) in Run 1, immediate post-thaw viability (D0) was high across most media (94.5-99.2%) (**Figure 5A**). After overnight resting (D1), viability remained high for FBS10 (96.0%), CS10 (95.5%), and CS7.5 (93.8%), but declined significantly for CS5 (85.7%), CS2 (78.0%), and Stem-Cellbanker D0 (72.9%) (all p < 0.001) compared to FBS10. In Run 2, immediate post-thaw viability (D0) ranged from 92.3% to 98.6%, with SF-CFM D10 (93.2%, p=0.0033) and Bambanker D0 (92.3%, p=0.0268) showing significantly lower viability compared to FBS10 (98.1%) (**Figure 5B**). After overnight resting (D1), FBS10, CS10, and NutriFreez D10 maintained high viability (94.5-95.6%), while Bambanker D10 (89.6%, p<0.001), SF-CFM D10 (90.9%, p=0.0055), and Bambanker D0 (72.0%, p<0.001) showed significantly lower viability compared to FBS10.

The same viability data is presented as the calculated difference between D1 and D0 in **Figures 5C, D**. While both metrics are informative, the viability percentages at D1 proved to be the most discriminative for identifying media performance, as significant differences between media became most apparent after overnight resting. The viability loss calculation (D1-D0) further illustrates these differences.

In Run 1, Stem-Cellbanker D0, CS2 and CS5 exhibited an increased GM viability loss immediately at M0, with values of 23% (95% confidence interval [CI]: 15.8; 33.4), 15.8% (95% CI: 10.6; 23.6), and 12% (95% CI: 8.3; 17.3), respectively. Similarly, in Run 2, Bambanker D0 displayed a comparable pattern, showing a GM viability loss of 19.8% (95% CI: 15.7; 24.9). For the remaining media, the GM viability loss never exceeded 8% across various time points, indicating more consistent stability.

Cell recovery rates (**Figures 5E, F**) reinforced these findings. CS2 and CS5 (Run 1), and Bambanker D0 and SF-CFM D10 (Run 2) showed lower cell recovery, suggesting a greater impact on both viability and cell recovery after cryopreservation. In contrast, solutions like CS10, NutriFreez D10, and the reference FBS10 maintained viability and cell recovery with only minor variability at specific time points.

Overall, certain media (CS2, CS5, StemCB D0, and Bambanker D0) demonstrated higher viability loss and reduced recovery, whereas others remained stable and comparable to the reference FBS10, indicating their suitability for long-term preservation.

These viability and recovery data informed the selection of media for subsequent analyses. Based on these results, all media with less than 7.5% DMSO were not further evaluated after the 3-month time point. Additionally, SF-CFM D10 was excluded due to its short post-thawing shelf-life, which made it less practical for use.

Having established these baseline parameters, we proceeded to examine the preservation of specific immune cell populations.

### 3.2 PBMC immunophenotyping and multivariate analysis

To comprehensively assess the long-term impact of different cryopreservation media on PBMC subpopulations, we performed a detailed immunophenotyping analysis at the 24-month (M24) time point, focusing on the most promising media identified in our earlier viability and functionality assessments. At M24, the abundance of PBMC subpopulations was assessed using samples of the 11 donors stored in the two most promising media, that is, CS10 and NutriFreez D10. These two media were selected because they had demonstrated the greatest similarities with the reference medium in all previous evaluations and thus were serious candidates to serve as replacement media. PBMC samples cryopreserved in a DMSO-free medium (Stem-Cellbanker D0) were included as a negative control.

**Figure 6:**
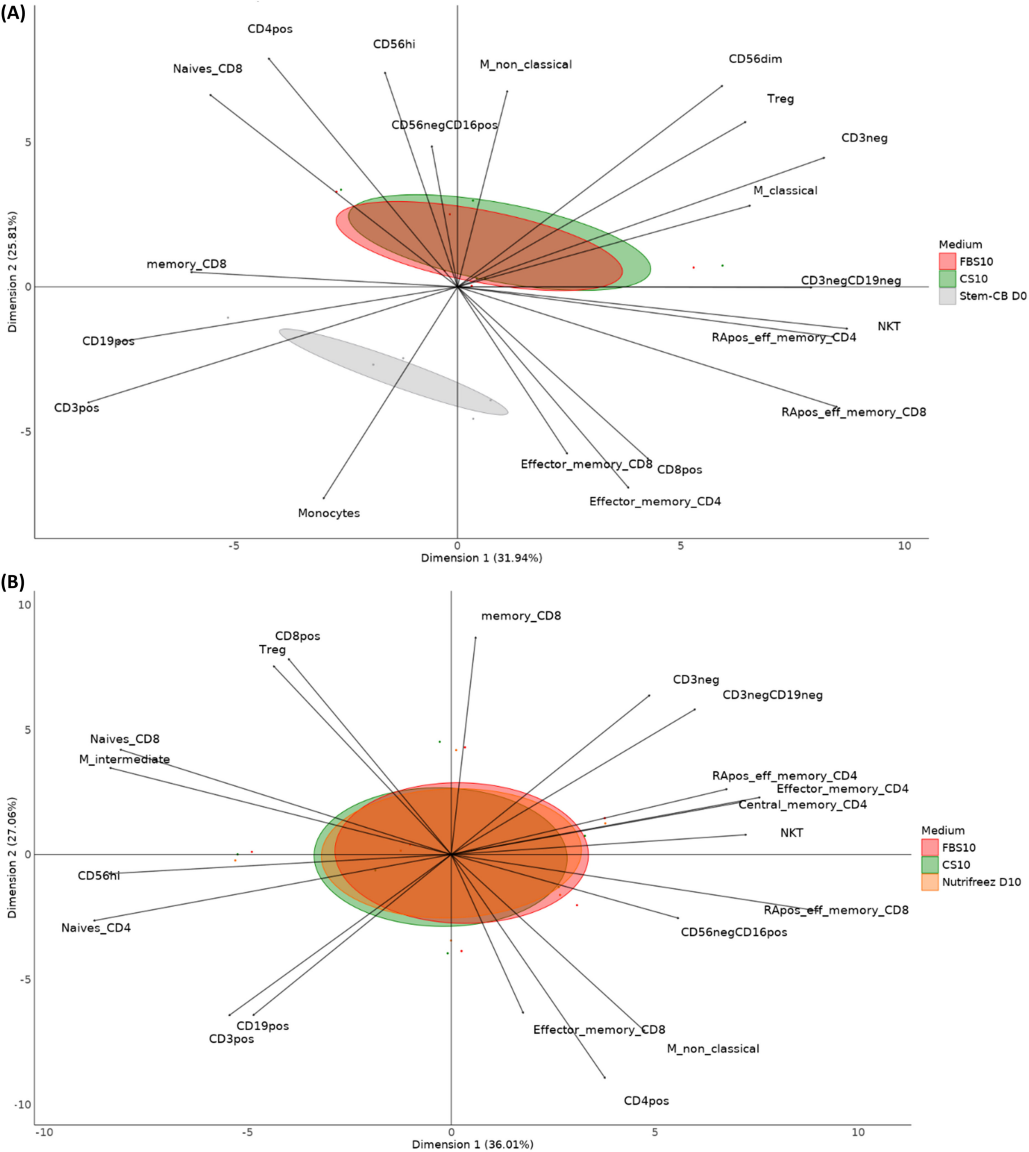
Principal component analysis (PCA) of immune cell populations in different media conditions in Run 1 and Run 2. Display of the various immune cell populations’ distribution across different freezing media conditions. Key cell populations include CD4+ T cells, CD8+ T cells (naïve, memory, effector memory), regulatory T cells (Tregs), monocytes (classical and nonclassical), NK cells (CD56dim, CD56high, CD56−CD16+), NKT cells, B cells (CD19+), and others. The PCA plot illustrates the separation and clustering of cell populations along two principal components. The first PCA compared the results of five donor samples from Run 1 **(A)** frozen in FBS10, CS10, or Stem-Cellbanker D0. The second PCA **(B)** compared the results of six donor samples frozen in FBS10, CS10, or NutriFreez D10 for Run 2.

To analyze the complex relationships between different cryopreservation media and their effects on immune cell subpopulations, multivariate analyses were performed on the phenotyping results–expressed as a percentage of the parent population–of 23 cell populations. **Figure 6** presents PCA plots comparing different freezing media conditions across two runs.

The plot from Run 1 compares CS10 and Stem-Cellbanker D0 to FBS10 and captures 57.7% of the variance in the data (3rd and 4th components respectively account for 17.4% and 11.5% of variability). FBS10 (red) and CS10 (green) distributions largely overlap, indicating similar impacts on immune cell profiles. However, Stem-Cellbanker D0 shows a distinct separation, particularly in CD56dim NK cells, monocytes, and effector memory T cells, suggesting a unique impact on these cell subsets. Building on these findings, we extended our analysis to Run 2, which included an additional promising cryopreservation medium. The plot from Run 2 compares CS10 and NutriFreez to FBS10 and captures over 60% of the variance in the data (3rd and 4th components respectively account for 12.8% and 8.8% of variability). The overlapping distributions indicate no significant differences in immune cell profiles among these media, suggesting CS10 and NutriFreez are equivalent to FBS10 in maintaining immune cell population integrity during freezing (**Figure 6**).

**Figure 7:**
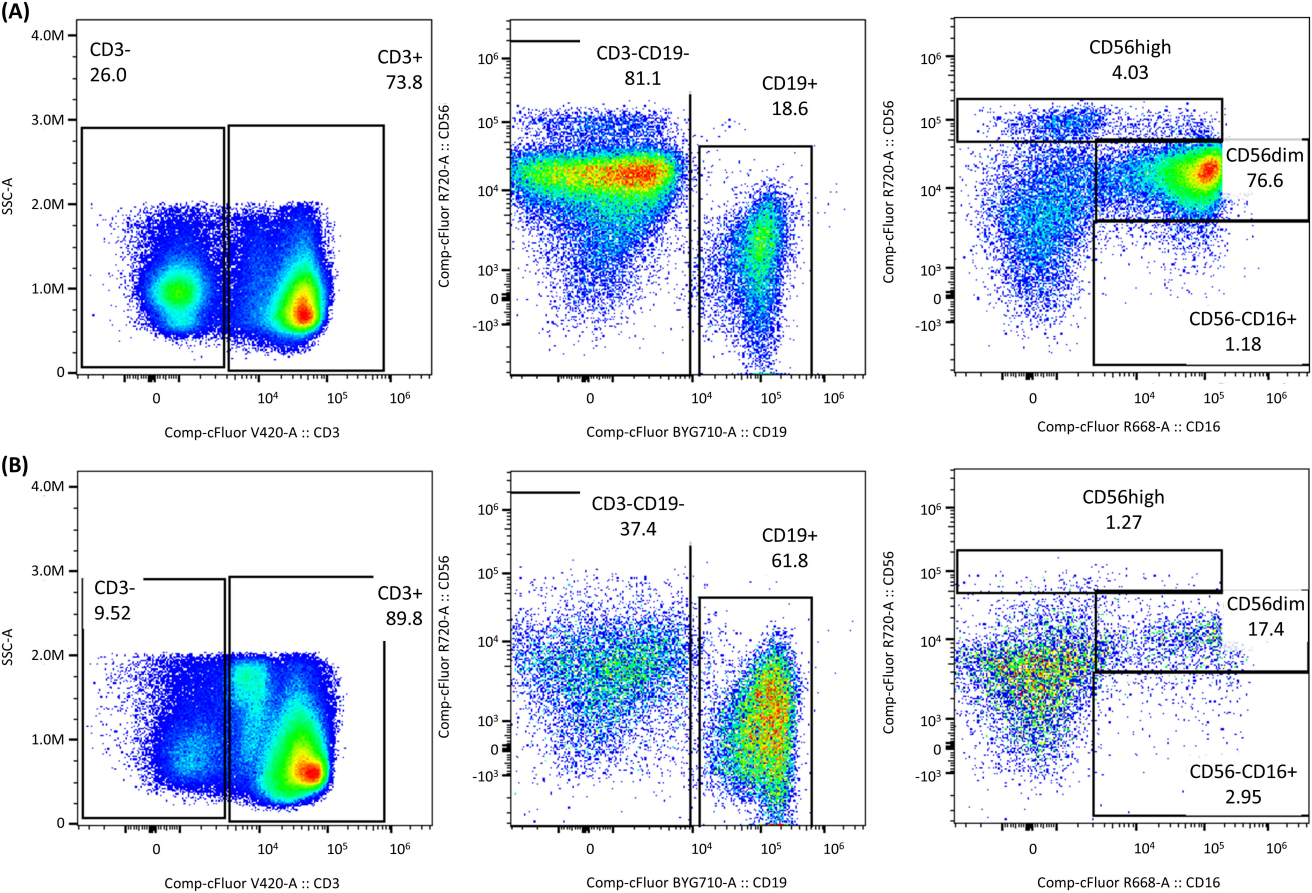
Impact of DMSO-free cryopreservation on NK cell subpopulations. Differences observed between PBMCs stored in **(A)** FBS10 (reference media) and **(B)** DMSO-free media (Stem-Cellbanker D0), focusing on NK cell subpopulations. Significant changes were observed in the proportions of CD3+, CD19+, and NK cell subpopulations. For instance, CD56dim NK cells reduced from 76.6% in FBS10 to 17.4% in DMSO-free media, while CD19+ cells increased from 18.6% to 61.8%.

**Figure 7** illustrates the differences observed between PBMCs stored in FBS10 (reference media) and a DMSO-free media, focusing on NK cell subpopulations. Significant changes were observed in the proportions of CD3+, CD19+, and NK cell subpopulations. For instance, CD56dim NK cells reduced from 76.6% in FBS10 to 17.4% in DMSO-free media, while CD19+ cells increased from 18.6% to 61.8%.

These results demonstrated a significant alteration in the cellular profile when PBMCs were frozen without DMSO, impacting the distribution and prevalence of different immune cell populations. Such changes could have substantial implications for downstream analyses and interpretations of immune responses in cryopreserved samples.

After assessing the impact of cryopreservation on the distribution of cellular subpopulations, we next investigated its impact on the functional capacity of memory B cells and T cells.

### 3.3 Memory B cell response

Having observed no significant differences in viability and cell numbers post-thawing among the 10% DMSO freezing medium candidates, we sought to evaluate the functionality of B cells cryopreserved in these media compared to PBMCs in the reference medium. To this end, a memory B cell FluoroSpot assay was performed, quantifying antigen-specific memory B cells after a 5-day polyclonal stimulation, during which B cells differentiate into antibody-secreting cells.

**Figure 8:**
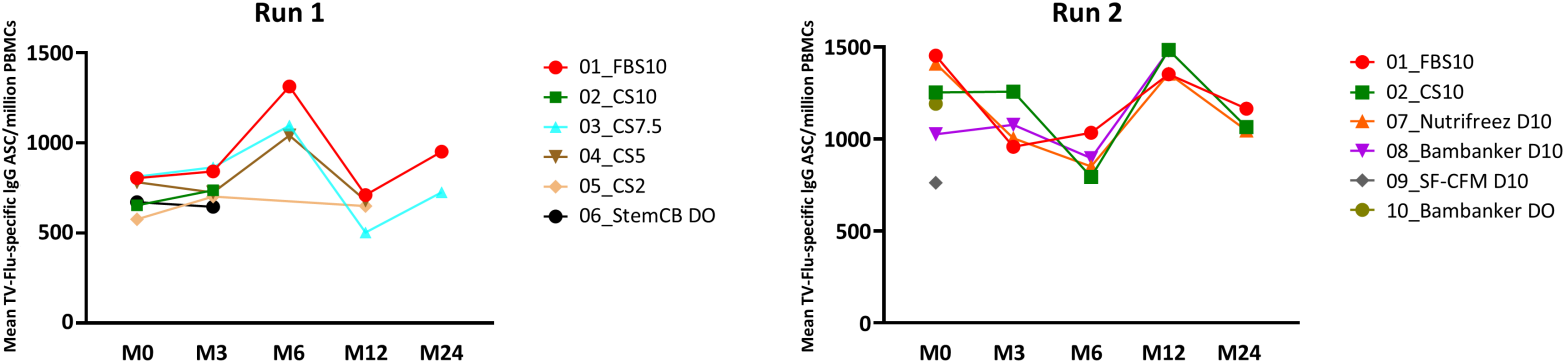
Comparable preservation of antigen-specific memory B cell responses in PBMCs cryopreserved with FBS10, CS10, and NutriFreez D10 as revealed by FluoroSpot assay. Longitudinal analysis of geometric means of VGT-specific IgG ASC/million PBMCs across different cryopreservation media at various time points (M0, M3, M6, M12, M24) over 24 months. Data from Run 1 (left panels, GM of five donors) and Run 2 (right panels, GM of six donors) are presented. The trends across time show the variability in the frequency of TV Flu-specific MBC depending on the freezing medium used. It is to be noted that evaluation of some media was abandoned after M0, M3, or M12.

In Run 1, we observed a consistent frequency of antigen-specific IgG memory cells across all media, with FBS10, CS10, and CS7.5 demonstrating particularly homogeneous responses and stability over time. PBMCs cryopreserved in CS2, CS5, and Stem-Cellbanker D0 showed slightly lower individual values (**Figure 8**). Run 2 yielded similar results, with stable frequencies noted for FBS10, CS10, and NutriFreez D10, while Bambanker D0, Bambanker D10, and SF-CFM D10 showed lower responses and increased variability. Notably, the heterogeneity of results observed with some media appeared to be primarily attributable to donor-specific factors rather than the cryopreservation media themselves.

It is worth noting that the decreased cell viability and cell number observed in some media might not have significantly impacted this read-out, as we cultured the same number of viable cells *in vitro* for 5 days.

### 3.4 T cell functionality

To comprehensively evaluate T cell functionality across different cryopreservation media, we employed three complementary assays: bulk cytokine secretion analysis, single-cell FluoroSpot assay for IFN-γ and IL-4 production, and multiparameter intracellular cytokine staining.

#### 3.4.1 Bulk cytokine secretion profiles in stimulated PBMC supernatants

**Figure 9:**
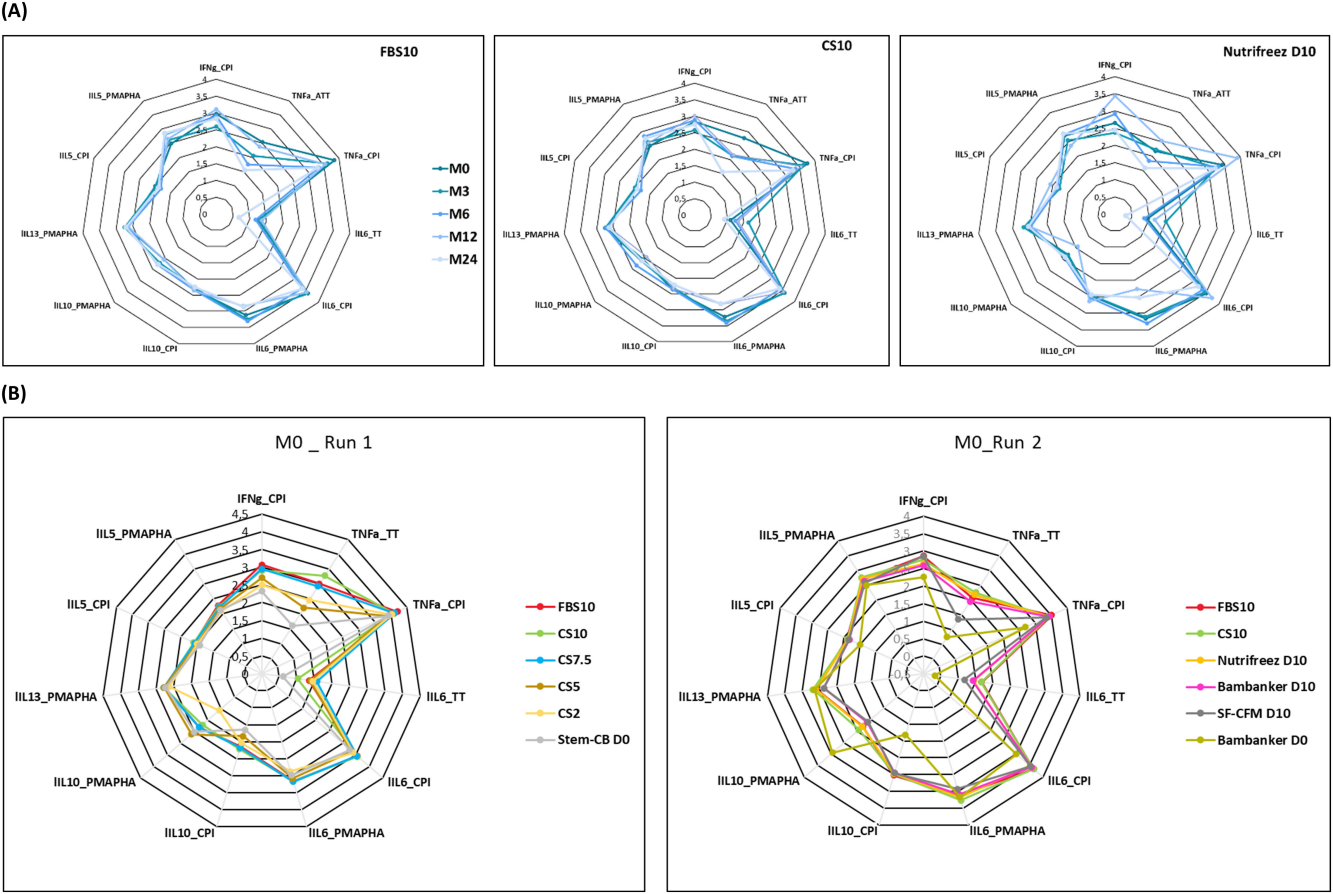
Cytokine profiles of PBMCs across different cryopreservation media and time points. **(A)** Spider charts illustrating cytokine-antigen combinations in PBMCs cryopreserved in FBS10, CS10, and NutriFreez D10 over 24 months (M0-M24, darker to lighter blue). Data represent mean values from 11 donors (FBS10, CS10) or 6 donors (NutriFreez D10). **(B)** Comparison of cytokine profiles at M0 for Run 1 (left, n=6) and Run 2 (right, n=5). Run 1 compares FBS10, CS10, CS7.5, CS5, CS2, and Stem-Cellbanker D0. Run 2 compares FBS10, CS10, NutriFreez D10, Bambanker D10, SF-CFM D10, and Bambanker D0. Colors represent different media as per legend. Analyzed combinations include IFN-γ, TNF-α, IL-6, IL-10, IL-13, and IL-5 responses to CPI, TT, and PMA/PHA stimulations.

To evaluate T cell functionality, we stimulated PBMCs with various antigens and measured a comprehensive panel of cytokine secretions in the supernatants. Our analysis included multiple cytokine-antigen combinations, including IFN-γ and TNF-α responses to CPI and ATT, as well as IL-6, IL-10, IL-13, and IL-5 responses to CPI, ATT, and PMA/PHA stimulations. **Figure 9A** presents spider charts displaying these cytokine profiles for PBMCs cryopreserved in FBS10, CS10, and NutriFreez D10. Each line on the chart represents a different time point, ranging from M0 (dark blue) to M24 (light blue), with lighter shades indicating later time points. Strikingly, the spider charts for these three media were highly similar, demonstrating consistent T cell responses across different cryopreservation solutions over the two-year period.

We observed a consistent decline in TNF-α levels following ATT stimulation over time across all three media (**Figure 9A**). However, this trend was uniform across FBS10, CS10, and NutriFreez D10, suggesting that it was not media-specific but rather a general effect of long-term cryopreservation.

In contrast, **Figure 9B** highlights the differences observed at M0 between DMSO-containing and DMSO-free media. The polygonal shapes corresponding to the DMSO-free media (Stem-Cellbanker D0 in Run 1 and Bambanker D0 in Run 2) were positioned closer to the center of the spider chart, indicating decreased cytokine secretion compared to DMSO-containing media.

It is important to note that we used equal numbers of viable cells in culture for each condition, which helps to control for differences in cell viability and quantity between media. This approach allows us to attribute observed differences more directly to the functional capacity of the surviving cells rather than to differences in cell numbers.

Overall, this analysis demonstrates that FBS10, CS10, and NutriFreez D10 maintain comparable T cell functionality over long-term cryopreservation, as evidenced by similar cytokine secretion profiles. In contrast, a trend toward decreased cytokine release was observed in DMSO-free media, though differences did not reach statistical significance, likely due to substantial inter-donor variability. This trend suggests a decrease in T cell functionality in DMSO-free conditions. These findings underscore the importance of DMSO in preserving T cell function during long-term cryopreservation and highlight the potential of CS10 and NutriFreez D10 as viable alternatives to FBS10. While bulk cytokine analysis provides valuable insights, we sought to complement these findings with single-cell resolution techniques.

To further characterize T cell responses at the single-cell level, we employed the FluoroSpot assay, which offers enhanced sensitivity for detecting cytokine-secreting cells.

#### 3.4.2 Single-cell analysis of IFN-γ and IL-4 production (FluoroSpot assay)

**Figure 10:**
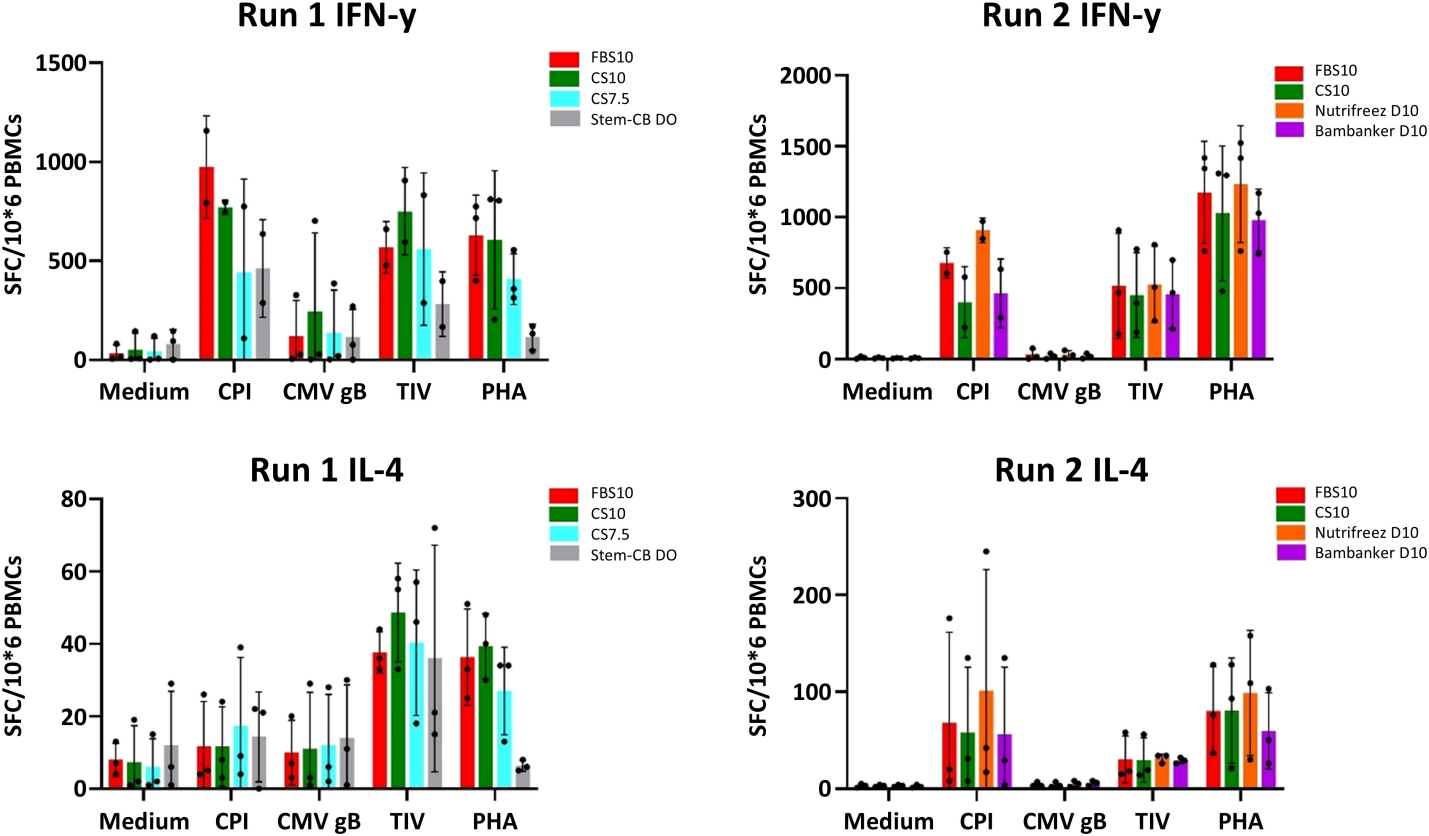
Comparable T cell functionality across most cryopreservation media with notable differences in Stem-Cellbanker D0 (Run 1) as measured by IFN-γ and IL-4 FluoroSpot assay. FluoroSpot assay results for IFN-γ (upper panels) and IL-4 (lower panels) secreting T cells from PBMCs cryopreserved in different media. The left panels show data from Run 1, and the right panels show data from Run 2. PBMCs were stimulated with medium (negative control), CPI, CMV gB, TIV, and PHA (x-axis). The y-axis represents the number of spot-forming cells (SFC) per million PBMCs. Bar charts depict the mean ± SD for 3 donors in each run, while individual donor results are shown as dots. Cryopreservation media are represented by different colors: Run 1: FBS10, CS7.5, CS10, and Stem-Cellbanker D0, Run 2: FBS10, NutriFreez D10, CS10, and Bambanker D10. This assay was performed at the M6 time point.

T cell functionality in response to various antigens (CPI, TIV, CMV gB, and PHA) was assessed using the highly sensitive FluoroSpot single-cell assay, which is particularly valuable for qualifying new FBS lots due to its ability to detect non-specific background noise. This approach complements bulk cytokine secretion measurements in the supernatant and helps evaluate the impact of different PBMC-freezing media. This assay was only performed at the M6 time point. The most notable difference was observed in response to non-specific stimulation with PHA, where PBMCs stored in Stem-Cellbanker D0 during Run 1 showed a reduction in the number of IFN-γ and IL-4 secreting cells. This reduction was more pronounced than that observed for specific antigen stimulations, such as CMV gB or CPI. Notably, the other freezing media did not induce any discernible non-specific T cell responses in unstimulated condition compared to the reference medium. In this study, no significant difference in IFN-γ and IL-4 secretion was observed in cells cryopreserved in the candidate media, with the exception of Stem-Cellbanker D0 (Run 1), when compared to the reference media (**Figure 10**). To further characterize T cell responses at the single-cell level and assess a broader range of cytokines, we employed multiparameter intracellular cytokine staining.

#### 3.4.3 Multiparameter single-cell analysis: intracellular cytokine staining

To evaluate the impact of freezing media on rare antigen-specific T cells, PBMCs stored in different media were thawed 6 months after the 2 effective years (M30), stimulated with peptides (6 hours) or proteins (CPI or Pokeweed lectin, for 6 or 9 hours), and analyzed for phenotypic markers and intracellular cytokines.

**Figure 11:**
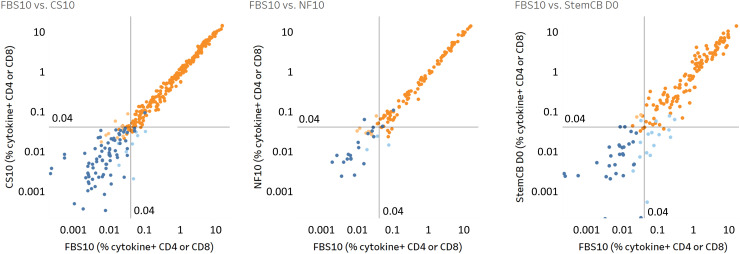
High correlation of antigen-specific T cell responses across different cryopreservation media demonstrates comparable preservation of T cell functionality. Comparison of antigen-specific T cell responses in PBMCs stored in FBS10 (reference medium) versus CS10 (left), NutriFreez D10 (middle), and Stem-Cellbanker D0 (StemCB D0; right). PBMCs were thawed, stimulated with CEFX (6 h) or CPI/PWM (6 or 9 h), and analyzed for various T cell subsets (IFN-γ+, IFN-γ+CD107a+, IFN-γ+granzyme+, IFN-γ+perforin+, IL-2+, IL-4+, IL-17A+, MIP-1β+, and TNF-α+ in CD4/CD8 T cells). Each point represents the percentage of a specific T cell subset. Response classification is color-coded: dark orange indicates responders in both media (R/R), dark blue shows non-responders in both media (NR/NR), light orange represents non-responders in FBS10 but responders in test medium (NR/R), and light blue denotes responders in FBS10 but non-responders in test medium (R/NR). Positive responses were determined by Fisher’s exact test. The analysis included 416 data points each for FBS10 and CS10 (from eight donors), 156 data points for NutriFreez D10 (from three donors), and 208 data points for Stem-Cellbanker D0 (from four donors).

**Figure 11** illustrates the responses of various T cell subsets (IFN-γ+, IFN-γ+CD107a+, IFN-γ+granzyme+, IFN-γ+perforin+, IL-2+, IL-4+, IL-17A+, MIP-1β+, and TNF-α+ CD4 and/or CD8 T cells) as percentages of specific cell populations. Scatter plots compare these responses from PBMCs stored in FBS10 with those in CS10, NutriFreez D10, and Stem-Cellbanker D0.

Pearson’s correlation analysis revealed strong correlations between the new media and FBS10 (CS10: r=0.994; NutriFreez D10: r=0.996; Stem-Cellbanker D0: r=0.951, all p<0.0001), indicating minimal impact on antigen-specific T cell responses across different media.

To define positive responses relative to the unstimulated control and assess potential loss of positive responses, a one-sided Fisher’s exact test was performed on ICS data, adjusted for multiple testing using FDR (*α*=0.001). Results are summarized in **Figure 11** and **Table 1**: CS10 showed 96% agreement (R/R or NR/NR) with 4% disagreement, NutriFreez D10 had 94% agreement with 6% disagreement, and Stem-Cellbanker D0 showed 91% agreement with 9% disagreement. Both the scatter plots and agreement counts demonstrate minimal impact of different freezing media on T cell functionality.

An analysis combining cellular integrity and T and B cell functional markers was conducted to identify patterns across all measured parameters.

### 3.5 PCA-based comparative assessment of freezing media integrating cellular integrity and functional markers

**Figure 12:**
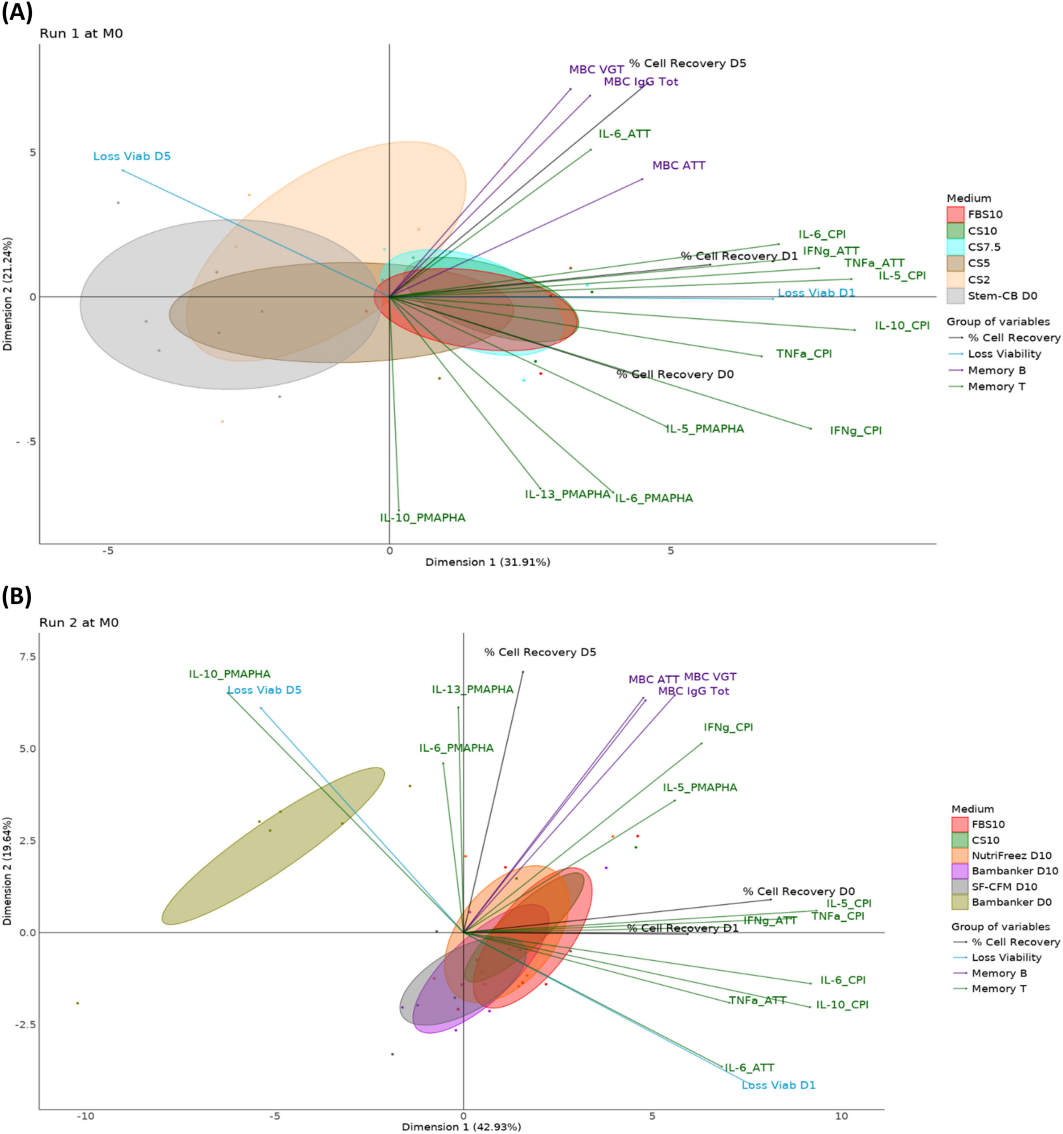
PCA reveals CS10 and NutriFreez D10 as most similar to FBS10 in preserving PBMC integrity and functionality. Run 1 **(A)** compares freezing media FBS10, CS10, CS7.5, CS5, CS2, and Stem-CB D0, while Run 2 **(B)** compares FBS10, CS10, NutriFreez, Bambanker D10, SF-CFM D10, and Bambanker D0. For both runs, the PCA graphs represent the M0 time point. Different media conditions are color-coded as indicated in the legend. Each ellipse represents the 95% confidence interval for the respective media condition, illustrating the spread and central tendency of the cell population data points within each group. Immune cell populations and functional markers are projected as vectors from the origin. The direction and length of each vector indicate the contribution and variance of each parameter along the principal components. These vectors are categorized and color-coded into four groups: % Cell recovery (dark gray), viability loss (blue), Memory B cell functionality (violet), and T cell functionality (orange).

To comprehensively compare different freezing media over time, we performed a PCA incorporating 20 parameters, including viability loss, cell recovery, and B and T cell functionality data (**Table 2**). **Figure 12** presents the PCA graphs, with each parameter category represented by a different color. This analysis was conducted for each time point and each run, enabling a comprehensive comparison of the different freezing media over time.

The PCA plots illustrate the separation and clustering of functional markers along two principal components. The plot from Run 1 compares samples from five donors preserved in FBS10, CS10, CS7.5, CS5, CS2, or Stem-Cellbanker D0 and captures 53.1% of the variance in the data (3rd and 4th components respectively account for 13.4% and 9.9% of variability). The plot from Run 2 compares samples from six donors cryopreserved in FBS10, CS10, NutriFreez D10, Bambanker D10, SF-CFM D10, and Bambanker D0 and captures 62.9% of the variance in the data (3rd and 4th components respectively account for 16.0% and 4.5% of variability).

The PCA analysis revealed distinct differences between freezing media and their effects on cell viability, recovery, and functionality. In Run 1, the reference medium (FBS10) showed relatively tight clustering, indicating consistent results across the measured parameters. CS10 clustered similarly to FBS10, while CS7.5 displayed slightly higher variability. In both runs, media with less than 7.5% DMSO (CS2, CS5, Stem-Cellbanker D0, and Bambanker D0) exhibited significant shifts along Dimension 1, primarily due to lower viability and recovery rates. This lower PBMC quality influenced the immunological responses of frozen cells. CS10 and NutriFreez D10 clustered most similarly to FBS10. SF-CFM D10 and Bambanker D10 showed a slight shift along Dimension 2, linked to T and B cell functionality. The DMSO-free Bambanker D0 was isolated, mainly due to poor viability and recovery rates. Overall, CS10 and NutriFreez D10 emerged as the media most similar to the reference FBS10 in preserving cellular integrity and functionality.

**Figure 13:**
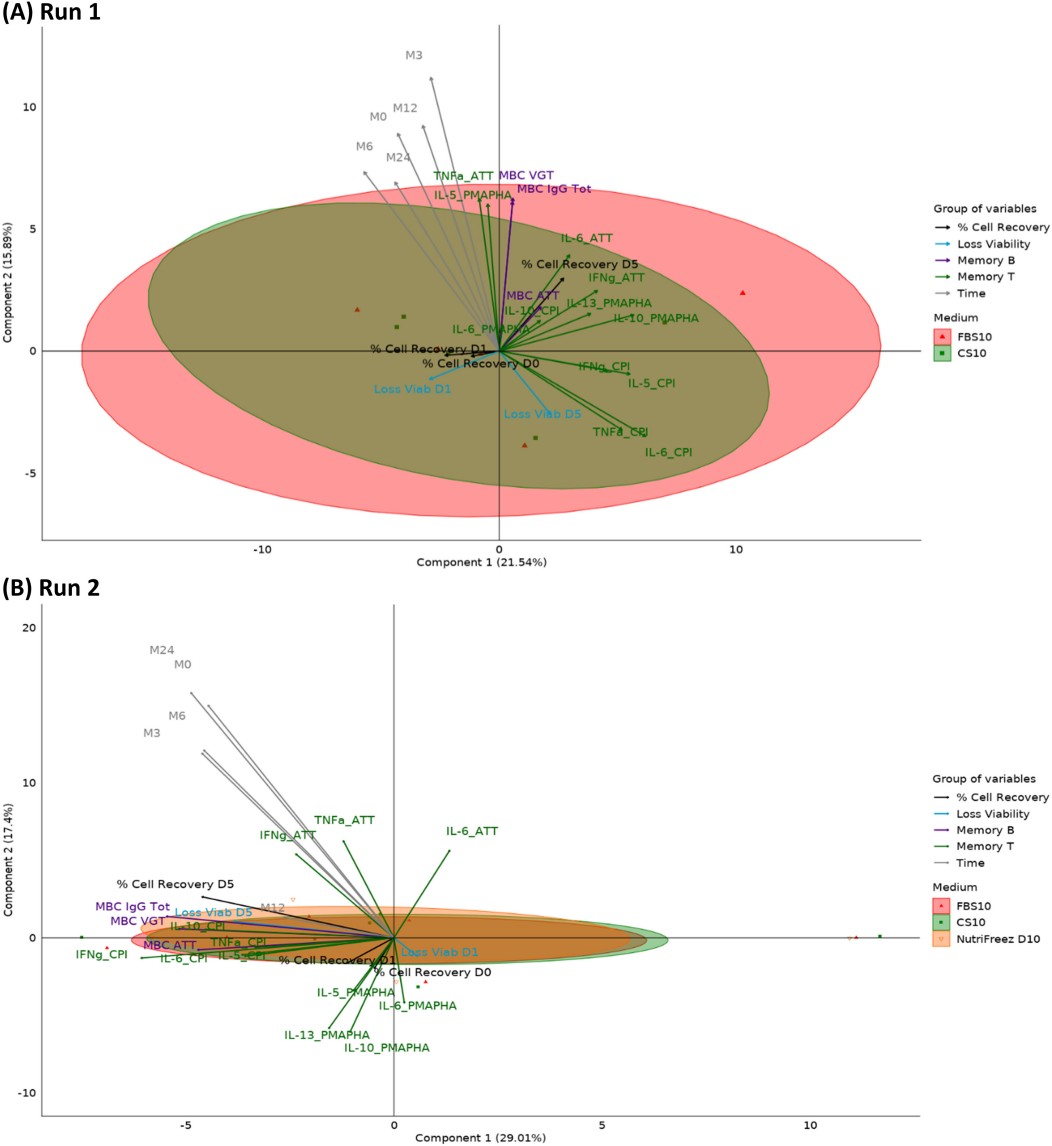
PARAFAC analysis of immunological readouts across cryopreservation media and time points. PARAFAC analysis of PBMCs cryopreserved in different media over multiple time points. **(A)** Run 1: FBS10 vs CS10 (n=5 donors). **(B)** Run 2: FBS10, CS10, and NutriFreez D10 (n=6 donors). Plots show the first two PARAFAC components. Ellipses represent 95% confidence intervals for each medium. Vectors indicate the contribution of 20 immunological readouts, categorized and color-coded as: % cell recovery, viability loss, Memory B cells, Memory T cells, and Time. Data from M0, M3, M6, M12, and M24. Axes show the percentage of variance explained by each component.

To further analyze the complex interactions between cryopreservation media, time points, and immunological readouts, we employed PARAFAC. This multiway data analysis technique extends traditional two-dimensional methods like PCA to handle three or more dimensions simultaneously. In our case, PARAFAC allowed us to integrate donor variability, multiple time points, and various immunological parameters into a single model. This approach provides a more comprehensive view of how different cryopreservation media perform across time and across multiple functional readouts. PARAFAC was performed only for the media for which data from the 20 immunological readouts were available at all time points (M0, M3, M6, M12, and M24), that is, FBS10 and CS10 for Run 1 and FBS10, CS10, and NutriFreez D10 for Run 2 (**Figure 13**). In **Figure 13A**, the first two components explain 37.53% of the variance (Component 1: 21.54%, Component 2: 15.98%, Component 3: 15.5%), showing a high overlap between FBS10 and CS10, suggesting their interchangeability in preserving immune markers such as viability and memory cell responses. **Figure 13B** extends this comparison by including NutriFreez D10, where the first two components account for 46.41% of the variance (Component 1: 29.01%, Component 2: 17.4%, Component 3: 8.9%). All three media show a significant overlap, indicating that they are functionally similar in preserving immunological readouts across time. Notably, time does not appear to contribute significantly to the overall variability, reinforcing the conclusion that FBS10, CS10, and NutriFreez D10 are comparably effective for cryopreservation.

In conclusion, our comprehensive analysis reveals that CS10 and NutriFreez D10 perform comparably to FBS10 in preserving PBMC subpopulations over time, while DMSO-free media show significant alterations in cellular profiles. These findings have important implications for selecting appropriate cryopreservation media in immunological studies.

## 4 Discussion

This comprehensive study evaluated the long-term cryopreservation of PBMCs using various animal-protein-free media compared to the standard FBS-supplemented medium. Our results demonstrate that CryoStor CS10 and NutriFreez D10, both serum-free media containing 10% DMSO, maintain PBMC viability, recovery, and functionality comparable to the FBS-based reference medium for up to two years. Through comprehensive analyses including multivariate PCA of 20 parameters and detailed immunophenotyping, we confirmed that these serum-free media preserved both T and B cell functions throughout the entire storage period. These findings have significant implications for standardizing PBMC cryopreservation protocols in clinical and research settings, addressing both ethical concerns and practical limitations associated with FBS use.

The superior performance of media containing 10% DMSO was evident across multiple functional readouts, while media with lower DMSO concentrations showed compromised cell preservation despite manufacturers’ claims. DMSO-free formulations demonstrated significant viability loss and substantial alterations in cellular profiles, with major shifts in the proportions of lymphocyte subpopulations that would compromise downstream immunological analyses. These findings highlight the critical role of DMSO in maintaining not only cell viability but also the representative distribution of immune cell subsets during long-term cryopreservation.

To evaluate different media, we deliberately exposed cells to optimal but fragile culture conditions. Measuring PBMC viability after overnight resting rather than immediately after thawing provides a more accurate assessment of cell quality, as it is known that using cells directly after thawing causes more variable results (14–18). The resting period allows pre-necrotic and pre-apoptotic cells—those damaged during freezing/thawing but initially still membrane-intact—to complete the apoptotic process and die, reducing background noise in functional assays and increasing T cell responses (17).

From the first time point (M0, 3 weeks after freezing), we demonstrated that the loss of viability after overnight resting and after a 5-day polyclonal stimulation were the most informative parameters for evaluating cryopreservation media quality. Media containing less than 7.5% DMSO showed significantly higher post-resting viability loss, with cell viability decreasing to between 70% and 85% compared to over 90% viability for cells frozen in media containing at least 7.5% DMSO. While literature acknowledges that viability over 70% is required for conducting robust functional assays (19–21), our results showed that PBMC samples with the lowest viability consistently exhibited weaker immunological responses against antigens, supporting our strategy of using post-resting viability as a key criterion for media evaluation.

Our study design incorporated an adaptive approach to media selection. Based on early viability and functionality results, we discontinued testing of media with less than 7.5% DMSO after the 3-month time point. This decision was supported by the significantly lower post-thawing viability observed in these media. We also eliminated media that presented practical challenges for routine laboratory use, such as SF-CFM D10, which requires frozen storage and has a short post-thaw shelf-life. Further refinement of our media panel occurred at the 12-month mark, where we removed CS7.5 due to its impractical preparation process and Bambanker D10 due to differences in T cell functionality compared to the reference medium. This iterative selection process allowed us to focus our long-term analysis on the most promising candidates, CS10 and NutriFreez D10, which consistently performed comparably to the FBS-based reference medium throughout the two-year study period.

While the direct cost of chemically defined media such as CS10 (~$4.00/mL) and NutriFreez D10 (~$4.50/mL) is higher compared to the traditional FBS+DMSO mixture (~$1.50-2.50/mL), this apparent price difference must be considered within a broader operational, regulatory, and ethical context. The use of FBS-containing media incurs substantial hidden costs: extensive batch-testing requirements, qualification procedures, and import restrictions that can significantly delay research timelines. Both CS10 and NutriFreez D10 are cGMP-compliant, sterility-tested, and ready-to-use formulations that eliminate these challenges while offering superior standardization through their chemically defined, serum-free, and protein-free compositions. Furthermore, these media eliminate the ethical concerns associated with FBS harvesting, aligning with the 3Rs principle (Replacement, Reduction, Refinement) in laboratory animal welfare.

Several limitations of our study should be acknowledged. First, our evaluation focused on PBMCs from healthy donors, reflecting our primary interest in vaccine trial applications. Future studies should validate these findings in samples from different patient populations. Second, while our sample size (n=11) was sufficient to demonstrate significant differences between media, larger cohorts could provide additional statistical power for subgroup analyses. Third, the study was conducted in two runs due to practical limitations in cell numbers obtainable from individual blood donations. While this staggered approach was necessary to test all media conditions and allowed us to include the best-performing medium from Run 1 in Run 2, it meant using different donor samples for each run. However, the consistency of our results across both runs supports the robustness of our findings. Fourth, we observed considerable inter-donor variability, particularly in functional assays using specific antigens, where responses varied based on individual infection and vaccination histories. While this variability complicated data interpretation, we deliberately chose to include antigen-specific stimulations alongside mitogenic stimulations because they engage different signaling pathways and provide a more comprehensive assessment of T cell functionality relevant to vaccine responses. Fifth, while core measurements (viability, recovery, memory B cell functionality, and cytokine secretion) were performed at all time points and showed no time-dependent effects, some specialized assays (immunophenotyping, intracellular cytokine staining, and ELISPOT) were conducted at single time points. The stability observed in our longitudinal measurements supports the validity of these single time point analyses. Finally, independent validation by other laboratories would further strengthen our conclusions.

## 5 Conclusion

These findings have significant implications, both in improving the standardization of PBMC cryopreservation and addressing ethical considerations. This comprehensive, long-term study represents a significant step toward establishing xeno-free media as a new standard in PBMC cryopreservation for clinical trials and immunological research. Our extensive evaluation over a two-year period demonstrates that the CryoStor CS10 and NutriFreez D10 ready-to-use freezing media are robust alternatives to the conventional FBS+10% DMSO medium. These xeno-free options not only match the performance of FBS-containing media in terms of cell viability and recovery rates but also preserve cellular functionality for at least two years post-freezing. The depth and duration of this study, encompassing multiple time points and a wide array of functional assays, provide a solid foundation for the broader adoption of these xeno-free media. By addressing the ethical concerns associated with FBS use and offering consistent, batch-independent performance, CryoStor CS10 and NutriFreez D10 present a promising path toward standardization in PBMC cryopreservation protocols. While further validation in larger cohorts and diverse clinical settings would be beneficial, our findings strongly support the transition to these xeno-free alternatives. This shift has the potential to enhance the reproducibility and ethical standards of immunological studies and vaccine trials, marking a significant advancement in the field of cellular cryopreservation.

## Data Availability

The original contributions presented in the study are included in the article. Further inquiries can be directed to the corresponding author.
